# When Do Sexual Partnerships Need to Be Accounted for in Transmission Models of Human Papillomavirus?

**DOI:** 10.3390/ijerph7020635

**Published:** 2010-02-22

**Authors:** Heidi Muller, Chris Bauch

**Affiliations:** Department of Mathematics and Statistics, University of Guelph, Guelph, Ontario, N1G2W1 Canada; E-Mail: hmuller@uoguelph.ca

**Keywords:** HPV, sexual partnerships, sexually transmitted infection, cervical cancer, public health policy, pair model, transmission model, dynamic model, human papillomavirus, vaccine

## Abstract

Human papillomavirus (HPV) is often transmitted through sexual partnerships. However, many previous HPV transmission models ignore the existence of partnerships by implicitly assuming that each new sexual contact is made with a different person. Here, we develop a simplified pair model—based on the example of HPV—that explicitly includes sexual partnership formation and dissolution. We show that not including partnerships can potentially result in biased projections of HPV prevalence. However, if transmission rates are calibrated to match empirical pre-vaccine HPV prevalence, the projected prevalence under a vaccination program does not vary significantly, regardless of whether partnerships are included.

## 1. Introduction

### 1.1. Human Papillomavirus

Human Papillomavirus (HPV) infections are responsible for several gynecologic diseases, including abnormal cervical cytology, cervical dysplasia, cervical cancer and genital warts [1]. Cervical cancer accounts for nearly 10% of all cancers in women worldwide, making it the second most common cause of cancer in women [2]. However, the burden is also disparate across world regions, with cervical cancer incidence varying from over 30 per 100,000 women per year in Africa to under 10 per 100,000 women per year in developed countries [2]. Mortality rates for women who develop cervical cancer also vary dramatically across regions, from over 60% in Africa to about 30% in developed countries [2]. In Ontario, Canada, for instance (population: 13 million), about 500 women are diagnosed with cervical cancer each year and 140 women die from it [3].

About 75% of adults will have at least one type of HPV in their lifetime [1,3,4]. HPV DNA has been detected in up to 99.7% of invasive cervical cancers worldwide [5]. The association between HPV and cervical cancer is unique; no other major human cancer has a single necessary cause [6,7]. Cervical Cancer is the second most common malignant disease in women worldwide, and generally affects individuals at a younger age than other cancers do [8,9].

There are approximately 130 types of HPV, of which 30–40 are transmitted via sexual contact [1,10]. Many types of HPV are carcinogenic (high-risk) including types 16 and 18 [3,11,12]. Some types cause genital warts, while other types are benign [3,12]. The prevalence of carcinogenic HPV types seems to be higher than the prevalence of non-carcinogenic types [13–16].

Carcinogenic HPV types are highly prevalent in Ontario women, infecting about 1 in 4 women aged 20–24 years [17]. Although cervical cancer is the cancer most frequently associated with HPV, HPV is also implicated in many anal, perianal, vulvar, and penile cancers [18]. Most HPV infections do not cause disease; these genital tract infections are, usually, transient lasting a few months. However, carcinogenic types tend to persist, especially HPV 16 [1]. HPV types 16 and 18 are the cause of 60–72% of cervical cancers and types 6 and 11 cause genital warts [19]. Genital warts, although not malignant, are associated with significant psychosocial morbidity and typically require multiple physician visits for diagnosis and treatment [20,21].

HPV is unlike most other sexually transmitted infections (STIs), in that infection is not highly concentrated in small groups of highly sexually active people, referred to as “core groups” [11]. Rather, HPV is very common across the population, although incidence is consistently highest in sexually active women less than 25 years of age and drops off with increasing age, even when adjustments are made for number of sexual partners [3,12,22,23]. Decreasing age of sexual debut, increasing number sexual partners and/or concurrent partners, and shortening time interval between successive partnerships all contribute to increasing the risk of infection [12,22–24].

### 1.2. HPV Vaccination

A multivalent vaccine that protects against infection by types 6, 11, 16 and 18 is now available [3]. It has been approved for females aged 9 to 26 and is most effective when given before sexual debut. Vaccination programs have since been implemented in Canada and other countries [25–27]. A bivalent vaccine protecting against types 16 and 18 is also becoming available [28]. The efficacy of HPV vaccines has been shown to be exceptionally high, approaching 100% [11,29]. These trials have been international, blinded, randomized placebo-controlled clinical trials involving thousands of women without prior exposure to the virus and who are in their late teens and early 20s [1,11,23,25,30].

### 1.3. Mathematical Models of HPV

Many mathematical models have examined the transmission of HPV and impact of various possible vaccination programs. They have generally found that a vaccination strategy of pre-adolescent females is both highly effective and highly cost-effective, in terms of reducing HPV-associated health burdens such as cervical cancer incidence. These have included compartmental models, deterministic models and stochastic models [25,27,31–33]. Traditionally, transmission models assume homogeneous mixing in a one-sex or heterosexual population, which implies that sexual partnerships are instantaneous and of zero duration: each new sexual contact is with a new person [25,31–34]. This may be a reasonable simplification in some subgroups of highly promiscuous individuals, however, for the general population, this is rarely the case. More realistically, most partnerships have a nonzero length and there is also a positive time gap between partnerships [24]. Since STIs like HPV spread sexually, not including partnerships in a transmission model may potentially bias model projections [35,36].

### 1.4. Pair Models for Sexually Transmitted Infections

In an attempt to address this deficiency, a number of methods to incorporate sexual partnerships have been developed for modelling sexually transmitted infections, such as “pair models” and “pair approximations” [35,37,38]. In a pair model, the number of partnerships (pairs) is an explicit model variable and the process of partnership formation and dissolution is captured. For instance, Kretzschmar and Dietz discuss several variations on a pair model [39]. First they consider the spread of SI (Susceptible-Infected) infection in a pair model and compare two cases: one where every contact is instantaneous and with a new partner and a second pair model with nonzero partnership length. If partnership length is nonzero, they found that (1) infection prevalence may initially decrease even when R_0_ > 1, (2) the growth rate is lower and (3) the endemic equilibrium is higher, than in a model without partnership duration. It is also shown for models with nonzero partnership length that a single value of R_0_ can imply more than one possible epidemic growth rate and endemic equilibrium. (R_0_ is the basic reproductive ratio, *i.e.*, the average number of secondary infections produced by an infectious person in an otherwise susceptible population.) Therefore, R_0_ cannot be estimated from empirical data on prevalence of a sexually transmitted infection without additional information on partnerships. Kretzschmar and Dietz also found that the transmission dynamics and R_0_ are affected by the assumed partnership dynamics for HIV, in particular.

Similarly, Dietz and Hadeler developed a simple SIS (Susceptible-Infected-Susceptible) model with pair dynamics [36]. The transmission dynamics in their model depend on the contact rate within a pair and the duration of a partnership. They find that with a partnership separation rate that is sufficiently large, an endemic equilibrium can persist. They derive R_0_ to determine the minimum intervention efforts required to eradicate an infection.

These previous models show that common insights based on classical epidemiological theory using homogeneous (non-pair) mixing models may no longer be valid once pair dynamics are included [36,37]. The reason why pair dynamics can impact transmission can be seen in several examples. For instance, if two individuals are both susceptible to HPV and they remain in the same partnership, with no concurrent partners, then they can never be infected. If a partnership forms between a susceptible and an infected person, then for a highly transmissible pathogen, the susceptible person will quickly become infected. From that point on, as long as both partners remain monogamous, additional contacts are “wasted” from the point of view of the virus [35]. Moreover, if the infection is totally cleared by the immune system before the partnership ends then neither partner can transmit infection to their new partners. Such effects can slow down the spread of disease relative to what would occur in a homogeneous mixing model. On the other hand, if the turnover rate of partnerships is very high, then partnerships do not last very long and a newly infected individual may pass on infection to a new sexual partner before the virus clears, allowing transmission to continue.

### 1.5. Outline of Paper

In this paper, we develop and analyze a pair model for HPV transmission and vaccination, assuming a Susceptible-Infectious-Recovered-Susceptible (SIRS) natural history. We analyze special cases of the model, considering the limit as the duration of partnerships goes to zero, in order to assess the impact of not including partnerships on model predictions. While the model is simplified in many respects, a simplified model is well suited to our objective of illustrating the impact of including *versus* excluding partnerships in STI transmission models. The results and discussion appear in section 2, the methodology is described in section 3, and the conclusions are drawn in section 4.

## 2. Results and Discussion

**Figure 1.**
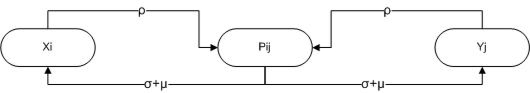
Schematic of pair dynamics. Single females, *X*_i_, and single males, *Y*_j_, form partnerships *P*_ij_ at a rate *ρ*, where *i,j* = S,I,R. These partnerships are destroyed either when the relationship breaks up (which occurs at a rate *⌠*) or when one partner dies (which occurs at a rate *μ*).

**Figure 2.**
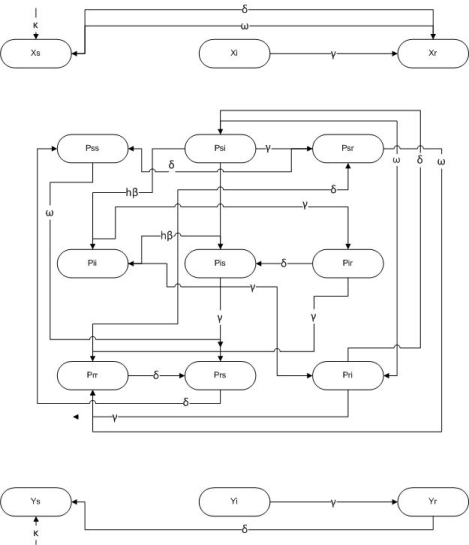
Schematic of infection dynamics. Females only are vaccinated, at a rate *⌉*. Infection can only occur within a partnership at a rate h*®*. Infected individuals recover at a rate γ and both natural and vaccine-derived immunity wane at a rate *δ*. Single females and males are recruited to the sexually active population at a rate /.

The HPV pair model describes single males and females forming monogamous sexual partnerships at constant rate *ρ* perunittime per capita. An individual is defined as single if they do not have a sexual partner at a given time. Within a given partnership, the number of sexual acts per year is *h*, and there is a probability of transmission from an infectious person to their susceptible partner of *β* per sex act. Transmission can only happen in a sexual partnership, and we consider transmission of vaccine-included carcinogenic HPV types only. A partnership breaks up at rate *σ* perunittime. Individuals recover to temporary immunity at rate *γ* perunittime and lose that immunity at rate *δ* perunittime. Males are recruited at rate *κ* males perunittime (same rate for females) and die at per capita rate *μ* perunittime. Susceptible females are immunized at rate *ω* perunittime. The model projects HPV prevalence over time. Prevalence is defined as the percentage of women infected with HPV at any given time; equilibrium prevalence is defined as the prevalence at the endemic equilibrium of the model equations. The model is represented through a system of ordinary differential equations (see Methods) that track the time evolution of partnerships and disease prevalence with or without vaccination. Additional details of the model appear in section 3, and baseline parameter values appear in Table 1. The model is represented schematically in Figure 1 (pair dynamics) and Figure 2 (infection dynamics).

**Figure 3.**
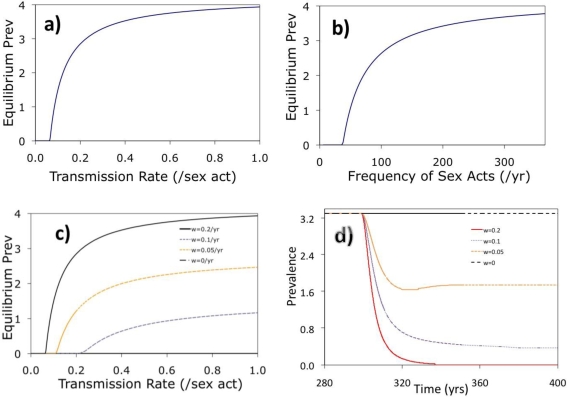
Transmission dynamics (equilibrium prevalence and time series) for various parameter values. Baseline parameters used are, μ = 1/15/yr, κ = mu*N/2, σ = 1/6/month, ρ = 33.66, γ = 1/yr, ω = 0/yr, h = 130/yr unless otherwise stated. Figure **3a** shows the impact of transmission rate, β on prevalence. Figure **3b** shows impact of average number of sex acts per year, h on prevalence, for and β = 0.4/act. Figure **3c** shows the impact of transmission rate, β, on prevalence for various vaccination rates ω, at baseline parameters. Vaccination rate include ω = 0/yr, ω = 0.05/yr, ω = 0.1/yr and ω = 0.2/yr. Figure **3d** shows a time series of prevalence for various vaccination rates introduced at year 300, with baseline parameters and β = 0.3/act.

This model system exhibits classical “threshold” behaviour. For instance, for sufficiently low values of the transmission probability per sex act, *β*, the disease prevalence is zero, but for values of β beyond a specific threshold value, equilibrium disease prevalence increases significantly and then appears to saturate as *β* increases further (Figure 3a). A similar pattern is seen with increasing average number of sex acts per year, *h* (Figure 3b). This “bifurcation” behaviour is a typical feature of epidemic models, where disease transmissibility must exceed a certain threshold in order for an endemic equilibrium to exist. In Figure 3a, the bifurcation point for the transmission probability per sex act *β* is between 0.05/act and 0.1/act. In Figure 3b, the bifurcation point for *h* is approximately between 25 and 50 sex acts per year. Vaccination has the effect of moving the bifurcation point to the right (higher threshold transmission rate *β*) and also lowering the prevalence for values of the transmission rate to the right of the threshold value of *β*, which is seen in Figure 3c. The effect of vaccination on the time evolution of prevalence is seen in Figure 3d, where increasing rates of vaccination cause more rapid declines in prevalence as well as lower long-term (equilibrium) prevalence. For sufficiently large coverage between ω = 0.1/yr and ω = 0.2/yr, the infection is eradicated. Eradication using relatively low vaccination rates occurs in STI transmission models where natural immunity is not durable [40]. Hence, this is not a surprising outcome in our model given that HPV natural immunity appears to be very short lived (Table 1, [14–16]). However, we emphasize that our model is a monogamous pair model, hence eradication of vaccine-included strains may be more difficult to achieve in real populations.

In order to examine how inclusion or exclusion of partnerships affects projected prevalence, the dynamics of the pair model can be explored for a range of possible values of the separation rate *σ*. In the limit as *σ* becomes very large, we recover the case of homogeneous (non-pair) mixing since the duration of partnership becomes zero and each new sexual contact is made with a new person. However, as *σ* increases, the formation rate *ρ* must also be increased to ensure that the total number of partnerships remains constant. Otherwise, if *ρ* were to be held constant, it is not obvious whether changes in prevalence as *σ* increases are due to changes in the duration of partnerships *per se* or just due to a decline in total number of pairs. The relationship between *ρ* and *σ* such that the number of pairs remains constant as *σ* changes can be obtained from the equilibrium solution of model differential equations (see Methods, Equation (14)). The case where *ρ* changes as *σ* changes such that the total number of pairs is constant and only the rate of partnership turnover is changing, will be referred to as the “*ρ* dependent” case. For the sake of comparison we also consider a “*ρ* fixed” case where *ρ* is held constant as *σ* changes, and the number of pairs therefore declines as *σ* increases.

**Figure 4.**
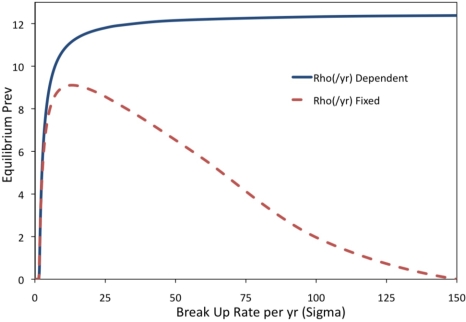
The impact of changes in the separation rate, σ, on percent infected for “ρ dependent” and “ρ fixed” cases. Baseline parameters to achieve 3% prevalence include μ = 1/15/yr, κ = μ*N/2, σ = 2/yr = 1/6/month, γ = 1/yr, h = 130/yr, β = 0.0737/act, ρ = 33.66/yr, and ω = 0. For the “ρ dependent” case, the equilibrium number of pairs was held constant at 8,875 according to equation (14) (see methods).

The impact of the partnership turnover rate in the “*ρ* dependent” case for a given value of the transmission rate *β* and frequency of sex acts *h* is very large (Figure 4). For small values of the turnover rate *σ*, the prevalence is zero because both partners clear the infection by the time they separate and seek new partners. However, as the turnover rate *σ* per year increases along the horizontal axis, a threshold is passed and the equilibrium prevalence begins to grow dramatically. For large enough values of *σ*, the prevalence levels off and reaches its maximum value of 12%. A plausible (realistic) value for *σ* in younger age classes is near the baseline value of *σ* = 2/year [24], which places dynamics in the regime where partnerships can impact prevalence. Hence, in this respect (for a given, fixed value of the transmission rate *β* and or number of sex acts per unit time *h*), homogeneous mixing models do not accurately capture transmission dynamics and will over-predict the prevalence of a sexually transmitted infection. The prevalence is also very sensitive to small changes in the turnover rate *σ* in realistic parameter ranges near the baseline value of *σ* = 2/year. In the “*ρ* fixed” case, prevalence is again zero for small values of *σ* (Figure 4). As *σ* increases, the prevalence again increases. However, unlike the “*ρ* dependent” case, the prevalence begins to decrease with increasing *σ*. This occurs because *ρ* is fixed as *σ* increases, therefore the total number of partnerships declines to a point that is not sufficient to support high prevalence.

**Figure 5.**
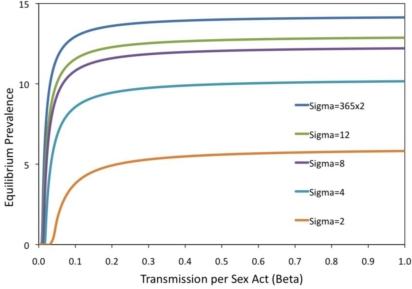
Impact of transmission rate, β, on prevalence for various turnover rates, σ for the “ρ dependent” case; baseline parameters μ = 1/15/yr, κ = μ*N/2, γ = 1/yr, h = 130/yr, ω = 0/yr were used. The equilibrium number of pairs was held constant at 8,875 according to equation (14) (see methods).

Figure 5 is analogous to Figure 3c, where the prevalence as a function of the transmission rate *β* is investigated. However, in this case, the way that the prevalence curve changes as the turnover rate *σ* changes is studied. The figure shows how increasing the turnover rate shifts the bifurcation point to the left (lower threshold transmission rate *β*) and also increases the equilibrium prevalence for all values of *β*. Prevalence changes most rapidly for smaller values of *β* just to the right of the bifurcation point. The baseline (realistic) value of *σ* = 2/year, the threshold is at approximately *β* = 0.3 per sex act. Figure 3 confirms that projected prevalence varies depending on whether partnership dynamics are included, for a broader range of parameters than were explored in Figure 4.

**Figure 6.**
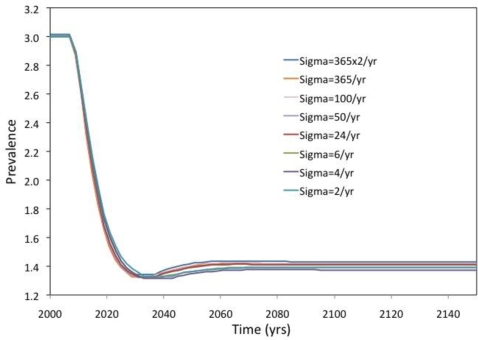
Time series of prevalence for various turnover rates, with vaccination introduced at year 300 at a rate ω = 0.05/yr. Baseline parameter values are as in the 3% prevalence case, μ = 1/15/yr, κ = μ*N/2, γ = 1/yr, h = 130/yr. The transmission rate per sex act, β, is calibrated for each turnover rate, σ to achieve 3% prevalence using the “ρ dependent” approach. The equilibrium number of pairs was held constant at 8,875 according to equation (14) (see methods).

Figure 6 presents the case where the transmission probability *β* is calibrated to achieve specified target prevalence in the pre-vaccine era, and the subsequent impact of vaccination is then projected. This is similar to how transmission models are often used in practice: the transmission rate parameters are calibrated until the prevalence in the model matches observed (empirical) prevalence pre-vaccine data. For Figure 6, a range of possible turnover rates *σ* are explored (with “*ρ* dependent” as in Figure 4). For each value of the turnover rate, the transmission rate β is calibrated such that the model predicts 3% prevalence at equilibrium of infection. (The value of 3% was chosen to reflect the approximate prevalence of high-risk HPV types 16 and 18.) Then, vaccination is introduced at a rate of *ω* = 5% per year in a population that is at the equilibrium prevalence of 3%, and the subsequent time evolution of prevalence is graphed. The projected time evolution is almost identical for all turnover rates *σ* analyzed, including both the relatively low baseline turnover rate of *σ* = 2/year as well as very high turnover rates of *σ* = 730/year (where partnerships last half a day) that approximate the pure homogeneous mixing case. From Figure 6, one can conclude that homogeneous models do not introduce inaccuracies, relative to a pair model, as long as β is calibrated to achieve specified target prevalence and the effect of vaccination on prevalence is the outcome of interest.

The analyses presented in Figures 3–6 were repeated for a higher 25% prevalence, as a form of sensitivity analysis, and the results and were found to be qualitatively similar (results not shown).

## 3. Methods

The model variables are *x*_i_ (the average (mean) number of single females of infection status *i* = S, I, R), *y*_j_ (the average (mean) number of single males of infection status *j* = S, I, R), and *P*_ij_ (the average (mean) number of pairs of infection status *i* = S, I, R and *j* = S, I, R). An individual is defined as single if they do not have a partner at a given time. The model equations resulting from the assumptions described at the beginning of Section 2 are given by

(1) $${\dot{x}}_{s}=\kappa -(\mu +\omega )x_{s}+\delta (X^{*}-x_{s}-x_{I})+(\mu +\sigma )(P_{SS}+P_{SI}+P_{SR})-(\Phi_{SS}+\Phi_{SI}+\Phi_{SR})$$

(2) $${\dot{y}}_{s}=\kappa -\mu y_{s}+\delta (Y^{*}-y_{s}-y_{I})+(\mu +\sigma )(P_{IS}+P_{SS}+P_{RS})-(\Phi_{SS}+\Phi_{IS}+\Phi_{RS})$$

(3) $${\dot{x}}_{I}=-(\mu +\gamma )x_{I}+(\mu +\sigma )(P_{IS}+P_{II}+P_{IR})-(\Phi_{IS}+\Phi_{II}+\Phi_{IR})$$

(4) $${\dot{y}}_{I}=-(\mu +\gamma )y_{I}+(\mu +\sigma )(P_{SI}+P_{II}+P_{RI})-(\Phi_{SI}+\Phi_{II}+\Phi_{RI})$$

(5) $${\dot{P}}_{SS}=-(2\mu +\sigma +\omega )P_{SS}+\delta (P_{RS}+P_{SR})+\Phi_{SS}$$

(6) $${\dot{P}}_{II}=-(2\mu +\sigma +2\gamma )P_{II}+h\beta (P_{SI}+P_{IS})+\Phi_{II}$$

(7) $${\dot{P}}_{IS}=-(2\mu +\sigma +\gamma +h\beta )P_{IS}+\delta P_{IR}+\Phi_{IS}$$

(8) $${\dot{P}}_{SI}=-(2\mu +\sigma +\omega +\gamma +h\beta )P_{SI}+\delta P_{RI}+\Phi_{SI}$$

(9) $${\dot{P}}_{IR}=-(2\mu +\sigma +\gamma +\delta )P_{IR}+\gamma P_{II}+\Phi_{IR}$$

(10) $${\dot{P}}_{RI}=-(2\mu +\sigma +\gamma +\delta )P_{RI}+\omega P_{SI}+\gamma P_{II}+\Phi_{RI}$$

(11) $${\dot{P}}_{RS}=-(2\mu +\sigma +\delta )P_{RS}+\omega P_{SS}+\gamma P_{IS}+\delta (P^{*}-P_{SS}-P_{II}-P_{SI}-P_{IS}-P_{IR}-P_{RI}-P_{RS}-P_{SR})+\Phi_{RS}$$

(12) $${\dot{P}}_{SR}=-(2\mu +\sigma +\omega +\delta )P_{SR}+\gamma P_{SI}+\delta (P^{*}-P_{SS}-P_{II}-P_{SI}-P_{IS}-P_{IR}-P_{RI}-P_{RS}-P_{SR})+\Phi_{SR}$$

where X^*^, Y^*^, and P^*^ are the equilibrium number of single females, single males and pairs, respectively (see equation (14)). The formation of new partnerships is described by the function:

(13) $$\Phi_{i,j}=\rho \frac{x_{i}y_{i}}{x_{S}+x_{I}+x_{R}+y_{S}+y_{I}+y_{R}}=\rho \frac{x_{i}y_{j}}{X^{*}+Y^{*}},\;\;i,j=S,I,R$$

where *ρ* is a parameter controlling the rate of partnership formation per unit time per capita. This function assumes that the rate at which males (respectively, females) of type *i* forms sexual partnerships with females (respectively, males) of type *j* depends on the proportion of females (respectively, males) in the population that are of type *j*. Note that the pair formation function is symmetric with respect to males and females.

The quantities X*, Y*, and P* are the number of single females, single males, and pairs at the equilibrium of partnership dynamics, respectively. These can easily be solved from the differential equations for pair dynamics in the absence of infection (not shown), yielding:

(14) $$(X*,Y*,P*)=\left(\frac{2\kappa (2\mu +\sigma )}{\mu (2\sigma +4\mu +\rho )},\frac{2\kappa (2\mu +\sigma )}{\mu (2\sigma +4\mu +\rho )},\frac{\rho \kappa }{\mu (2\sigma +4\mu +\rho )}\right)$$

This equation has been used to write down Equations (1)–(12) under the assumption that the pair dynamics have equilibrated in a population where infection and vaccination are introduced. Because these are the equilibrium number of total pairs and singles regardless of infection status, the expressions in Equation (14) are not dependent on the vaccination rate ω. Using equation (14) allows us to reduce the original system of equations by three dimensions by substituting in for *x*_R_, *y*_R_ and *P*_RR_ wherever they appear according to equation (14) and the following relations:

(15) $$x_{S}+x_{I}+x_{R}=X*$$

(16) $$y_{S}+y_{I}+y_{R}=Y*$$

(17) $$P_{SS}+P_{SI}+P_{SR}+P_{IS}+P_{II}+P_{IR}+P_{RS}+P_{RI}+P_{RR}=P*$$

Equations (1)–(12) were solved numerically in MATLAB using the built-in function ode23tb, an implementation of an implicit Runge-Kutta solver for systems of stiff differential equations, with a first stage that is a trapezoidal rule step and a second stage that is a backward differentiation formula of order two. Solving for *R*_0_ analytically is difficult due to the large dimensionality of the system of equations. Parameters are given in Table 1.

## 4. Conclusions

The first conclusion of this research is that models of STI transmission where partnerships of nonzero duration are explicitly included in the model yield projections that will generally differ from models where partnerships are not explicitly included (*i.e.*, homogeneous mixing is assumed). This echoes previous findings using pair models for SI and SIS infections [37,38]. In the current SIRS pair model, a sufficiently high partnership turnover rate (recovering the case of homogeneous mixing) was found to predict a higher prevalence than occurred at realistic values of partnership turnover rates.

However, if transmission rates are first calibrated to match observed prevalence and then used to predict the impact of vaccination, the predictions of these two types of models will be very similar, which may be surprising. Homogeneous (non-pair) models often use this type of calibration approach. This suggests that homogeneous mixing models may suffice for modelling STI transmission if they are calibrated to prevalence data and only being used to predict the impact of vaccination.

In practical terms, this means that the predicted impact of vaccination policies according to homogeneous (non-pair) mixing models cannot necessarily be discarded on grounds that sexual partnerships have not been accounted for. However, we note that there are important aspects of real-world sexual contact networks that we did not include in this analysis, such as concurrency (overlapping partnerships), stochasticity (random effects), sexual risk groups, and age structure. These elements have been shown to influence disease dynamics [37,38]. For instance, at low prevalence, stochastic effects can cause an infection to fade out, whereas the corresponding deterministic model cannot describe this process. However, a deterministic model was used because most previous models of HPV transmission are deterministic models, and our objective was to comment on the domain of validity of these previous HPV models. Our conclusions might also change if we were to compare homogeneous mixing models to a more realistic sexual partnership model. We also note that this model did not include a “core group” of highly sexually active individuals that sustain transmission and without whom the infection dies out. Further analysis can explore the role of highly active subpopulations in HPV transmission to determine whether they have a significant impact on transmission dynamics.

Finally, a more exhaustive exploration of parameter space may reveal parameter regimes where pair model projections of the impact of vaccination diverge from homogeneous mixing model projections, even when both models are calibrated to pre-vaccine prevalence. However, the biological plausibility of these parameter regimes would have to be considered. Rigorous analysis of the model equations, permitting a derivation of the basic reproduction number *R*_0_ for example, would facilitate understanding the relationship between model predictions and model parameters. However, derivation of *R*_0_ is difficult due to the large dimensionality of the system. Moreover, an expression for *R*_0_ would not provide much information about the transient nature of the solutions, and it is the transient solutions that are relevant to public health since transient solutions describe prevalence in the first few years or decades after a vaccination program is implemented.

Additionally, this model only considers infection from HPV types 16 and 18 since the vaccine is preventative for these types. However, there are 30–40 HPV types that are transmitted sexually and infection with certain types may inhibit or activate infection of other types. The interaction between HPV types, or the impact of potential highly multivalent vaccines, could also be studied in future work. Many of these limitations suggest areas for future research on this topic.

In summary, if homogeneous mixing models that neglect partnerships are used to assess the impact of vaccination programs or other interventions for sexually transmitted infections, it is important to understand how and whether the non-inclusion of partnership dynamics influences the projections of these models. This is particularly important if these models are used to inform public health policy.

## Figures and Tables

**Table 1.** Model parameters, values and sources.

Symbol	Definition	3% prevalence scenario	Source
ɛ	Vaccine efficacy	95%	[11]
μ	Rate at which individuals leave the age group of peak sexual activity /yr	1/15/yr	[2,3]
κ	Rate at which individuals are recruited into the age group of peak sexual activity /yr	μN/2	Derived (see Methods)
σ	Pair break-up rate /yr	2/yr	[27]
ρ	Pair formation rate /yr	33.66/yr	Derived using [27] (see Methods)
h	Number of sex acts /yr	130/yr	[31]
β	Transmission rate per sex act	0.073/act	[41,42], calibrated (see Methods)
ω	Rate at which females are vaccinated	0.05/yr	[42], calibration (see Methods)
γ	Infection clearance rate/yr	1/yr	[14,16,42]
δ	Natural immunity waning rate/yr	1/10/yr	[13,14,16]
